# The neural underpinnings of an optimal exploitation of social information under uncertainty

**DOI:** 10.1093/scan/nst173

**Published:** 2013-12-02

**Authors:** Ulf Toelch, Dominik R. Bach, Raymond J. Dolan

**Affiliations:** ^1^Berlin School of Mind and Brain, Humboldt University, 10117 Berlin, Germany, ^2^Bernstein Center for Computational Neuroscience Berlin, Charité – Universitätsmedizin Berlin, 10115 Berlin, Germany, ^3^Wellcome Trust Centre for Neuroimaging, University College London, London WC1N 3BG, UK, and ^4^Zurich University Hospital for Psychiatry, Switzerland

**Keywords:** social information, left inferior frontal gyrus, instrumental control, Bayes optimal integration, copy-when-uncertain, prefrontal cortex

## Abstract

Social information influences decision-making through an integration of information derived from individual experience with that derived from observing the actions of others. This raises the question as to which extent one should utilize social information. One strategy is to make use of uncertainty estimates, leading to a *copy-when-uncertain* strategy that weights information from individual and social sources based on their respective reliabilities. Here, we investigate this integration process by extending models of Bayes optimal integration of sensory information to a social decision context. We then use a key parameter of our behavioral model in conjunction with functional magnetic resonance imaging to identify the neural substrate that is specifically linked to the fidelity of this integration process. We show that individuals behave near Bayes optimal when integrating two distinct sources of social information but systematically deviate from Bayes optimal choice when integrating individual with social information. This systematic behavioral deviation from optimality is linked to activity of left inferior frontal gyrus. Thus, an ability to optimally exploit social information depends on processes that overcome an egocentric bias, and this regulatory role involves the left inferior prefrontal cortex. The findings provide a mechanistic explanation for observations wherein individuals neglect the benefits from exploiting social information.

## INTRODUCTION

Our decisions benefit from a constant stream of social information that includes the vicarious observation of the actions and outcomes of actions of others (Rendell *et al*., 2011). This type of influence provides a rich underpinning to much of human culture (Dean *et al*., 2012). Under a broad range of conditions, copying the actions of others results in the adoption of advantageous behavioral traits (Rendell *et al*., 2010). When observed actions, and their outcomes, are closely conjoined these stimulus response contingencies seem to be associatively learned using the very same prediction errors that underpin associative learning for non-social stimuli (Behrens *et al*., 2008; Burke *et al*., 2010; Heyes, 2012).

The influence of social information is often biased (Mesoudi *et al*., 2006) and modulated by context and individual predisposition (Alevy *et al*., 2007; Efferson *et al*., 2008; Toelch *et al*., 2009). This raises a fundamental question as to how individuals weight social and individual information in a decision-making context. One proposal is that a *copy-when-uncertain* strategy can account for situations wherein individually acquired information is imprecise. An unbiased account of this strategy suggests that each information source is weighted by its corresponding reliability with distinct information sources subsequently combined in a Bayes optimal (BO) manner. This type of integration process is evident in multi modal cue integration (Ernst and Banks, 2002; Alais and Burr, 2004; Knill and Pouget, 2004), as well as in joint decision making where participants appear to use a confidence metric to arrive at a BO response in perceptual tasks (Bahrami *et al*., 2010) cf. (Koriat, 2012).

**Fig. 1:**
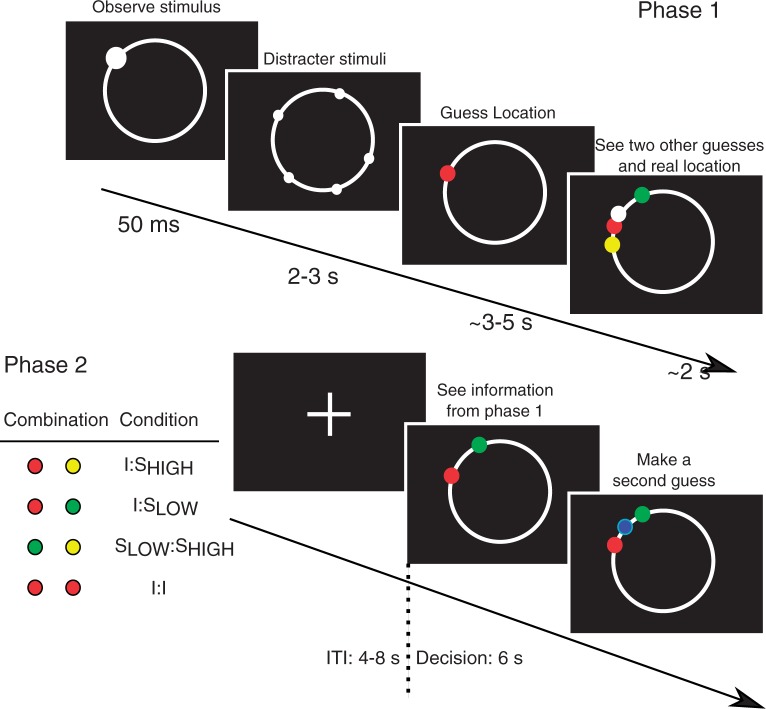
In phase 1 (120 trials), players assessed their own accuracy and the accuracy of two other players in a perceptual task where they had to guess the position of a briefly (50 ms) flashed stimulus. After observing the stimulus, several distracter stimuli appeared in quick succession in random locations along the circumference of the circle. During the whole time, players had to center their mouse pointer that was slowly moved by the computer in one direction. After this, players had to guess the position of the previously presented briefly flashed stimulus. They also saw the decisions of the two other players as well as the actual true position of the stimulus. In phase 2, there was no flashed target stimulus. Instead, players received information from phase 1. This was always a combination of two guesses consisting of either individual information (red) or social information (green and yellow). This led to four conditions (60 trials each); individual information paired either with high (*I*:*S*_HIGH_) or low (*I*:*S*_LOW_) accuracy social information, a condition with two social information pieces combined (*S*_LOW_:*S*_HIGH_) and a condition with only individual information (*I*:*I*). Based on this information, players then made a second guess (blue) as to the position of the stimulus but in this phase received no feedback.

When individuals have to integrate social and individual information, however, there appears to be a strong preference for individual information (Eriksson and Strimling, 2009; Morgan *et al.* 2012; Heyes, 2012) even when social information use is advantageous. Here, we propose that individuals over discount social information proportional to the reliability of their own information to account for the uncertainty inherent in social information (Rieucau and Giraldeau, 2011). We provide an experimental test for such a modified *copy-when-uncertain* strategy. The experimental manipulation of individual uncertainty estimates and with that the hypothesized differential use of social information opens up the possibility to index a neural basis for regulating the interplay of individual and social information. For this, we use functional magnetic resonance imaging (fMRI) in conjunction with behavioral modeling of player choices where the focus is on individual differences in social information use. In brief, our task required players to solve a perceptual task where they had to guess the location of a briefly flashed stimulus (Figure 1). In the first phase, players could assess their own accuracy as well as the accuracy of two players, one with high and one with low accuracy, through feedback as to the correct location (after they made a choice). In a second phase, conducted in an fMRI scanner, participants received information (individual and/or social) generated in the first phase and made a second guess on the position of the stimulus but now without receiving feedback on its actual true position. We modeled players’ choices using BO cue integration models. Parameters derived from these models were then used as regressors in a model-based fMRI analysis to identify brain areas critically linked to this modulation of choice.

## MATERIALS AND METHODS

### Participants

Twenty-nine participants (19–42 years, 17 females) were recruited via posters and paid €25 each for participation. All were right-handed and had normal or corrected-to-normal vision in the scanner. Participants reported no previous history of neurological or psychiatric illness. Before beginning, written instructions were given and participants were allowed to ask questions. Written informed consent was obtained from all participants. The procedures and questionnaires were approved by the Medical Ethics Review Committee of the Charité University Hospital (protocol number EA4/042/12).

### Experimental task

The task consisted of an initial training phase (20 trials) followed by two experimental phases (120 and 240 trials) and lasted all in all approximately 2 h. The training and first phase involved guessing the location of a stimulus that briefly flashed on the screen (Figure 1), whereas in the second phase players had to make a second guess based on their decisions from the first phase without seeing the stimulus again. The second phase was conducted inside the scanner. Participants were told via instructions that part of their remuneration (up to €9) depended on their decisions in both phases with an emphasis on the second phase (see Electronic Supplementary Material (ESM)). After the experiments, all participants received a fixed amount that amounted to almost the maximum possible.

### Training phase and first experimental phase

Each trial commenced by subjects pressing a key. After 1 to 2 s, a stimulus (filled circle) was briefly flashed (50 ms) on the outline of a pre-drawn circle on a computer screen (Figure 1). Immediately after stimulus presentation, distracter stimuli were displayed on the outline of the empty circle for 3 s. During this interval, the mouse pointer slowly moved from the center to the edge of the screen. Players were instructed to keep the pointer in the center of the screen or otherwise they would lose points. This manipulation prevented players from executing a saccade to the target location and fixate on the target location. Pilot studies showed that this difficulty level ensured inter individual variability in player accuracy. Participants then had to make a guess where on the outer circle the stimulus had appeared. Their guess was displayed as a small red circle.

In the first experimental phase, players saw, additional to their own choice, a green and a yellow circle representing the choices of two other players that were recorded in a pilot. The other players were selected from a pilot based on their accuracy with one player being more accurate than the other. To assess their own and other players accuracy, the subjects also saw a white circle representing the actual position of the flashed stimulus. Social players were not selected from the extremes of the available players from the pilot but had distinct average accuracies with a standard deviation of 15 degrees for one player and 7.3 degrees from the actual stimulus position (Figure 1).

### Second experimental phase

This phase was conducted within the environment of an MRI scanner. Each trial started after an inter trial interval during which only a small cross was visible on the center of the screen. In this phase, players viewed choices from trials from the first experimental phase in a randomized order. In each trial, players received two pieces of information to inform a second guess on where the stimulus was located but received no feedback on the actual position of the stimulus. There were four information pairs; their own guess paired with a guess of either the low accuracy or high accuracy demonstrator (*I*:*S*_LOW_ and *I*:*S*_HIGH_). Players also played trials where they received information from the two social players without having to integrate their own information (*S*_LOW_:*S*_HIGH_). They also received pairs that consisted of their own guesses alone (*I*:*I*). This was rendered possible as we presented players with a set of 60 stimuli in the first experimental phase that were in fact presented twice in randomized order. That is, that two guesses for each player were available for each stimulus location. Players were informed of this manipulation.

Thus, each condition entailed 60 trials. Players had 6 s to respond. The inter trial intervals (mean = 5 s; Min = 4 s; Max = 8 s) and order of trials were determined via randomization methods outlined in (Henson, 2007) to maximize the variance in the blood-oxygenation-level-dependent (BOLD) signal and at the same time reduce the duration of the experiment. In this phase, players used a response box to choose the assumed position of the stimulus moving a blue circle along the outer larger circle by using two of the response box buttons for left and right movement and one button to select (Figure 1). The blue circle started randomly near one of the two circles representing the information from phase 1 so that the movement duration was independent of condition. We compared reaction times between conditions using a generalized linear mixed model assuming an error structure from the gamma distribution family. We entered the four conditions as well as the distance the blue dot was moved in a round as dependent factors. We modeled player identity as a random effect on the intercept.

### Image acquisition and analysis

Whole brain T2*-weighted echo-planar imaging BOLD fMRI data were acquired with a Siemens Trio 3T (Siemens Medical Solutions, Erlangen, Germany) magnetic resonance scanner using a 12-channel head matrix coil, with 33 slices recorded in descending order (64 × 64 voxels; resolution 3 mm × 3 mm × 3.5 mm slices), a volume repetition time (TR) of 2000 ms, an echo time of 30 ms. The fMRI data were preprocessed and analyzed in an event-related manner with SPM8 software (Wellcome Trust Centre for Neuroimaging, London, UK). Preprocessing consisted of slice-time correction, spatial realignment, co-registration to the participants’ T1 image, normalization to Montreal Neurological Institute coordinates via the new segment procedure in SPM8, and spatial smoothing with a Gaussian kernel with a full width at half-maximum of 8 mm. We modeled each trial with a boxcar function at the trial onset with reaction time as length of the boxcar. For each of the four conditions, we created one regressor that was convolved with a canonical hemodynamic response function. To correct for motion-related artifacts, we modeled subject-specific realignment parameters as nuisance regressors. Additionally, we explored models where the distance between the two points was modeled as parametric modulator to detect neuronal correlates of uncertainty differences stemming from varying distance between the two points. These models did not yield any additional insights.

Linear contrasts of regression coefficients were computed at the individual subject level and then taken to a group-level random-effects analysis. We applied whole-brain family-wise error (FWE) correction for multiple comparisons on the basis of random field theory. We used a cluster FWE-corrected threshold of *P* < 0.05, on the basis of a voxel-wise threshold of *P* < 0.001 uncorrected. Subjects were scanned in two sessions of 120 trials interspersed by a T1-weighted structural scan.

### Behavioral model fitting

We modeled player decisions in the second phase using maximum likelihood methods to individual player data for decisions that involved an integration of different information (excluding the *I*:*I* condition). Players had two different types of information available namely (i) the distance between the two displayed choices (*D*_ini_) and (ii) their estimate of their own accuracy (acc_I_) and the accuracy of the social players (acc_S_). On the basis of this information, they made a second choice that usually deviated by a certain degree from their initial choice. The models differ with regard to whether and how these two information types are taken into account when determining the degree to which a players’ decision deviated from the initial choice (*D*_fin_). We chose the position of the individual information as reference to determine how far the player moved his second choice for the *I*:*S* conditions. For the *S*_LOW_:*S*_HIGH_ condition, we used the position of the low reliability player as reference. Note here that changing the reference does not impact on the outputs of the modeling process. We restricted our model analysis to cases where *D*_ini_ was below 30 degrees which led to an exclusion of 5.5% of the trials from the modeling process. Above this threshold, it was clear to players that only one point was reliable and that the other point had to be an erroneous choice stemming from a lapse of concentration during the game. Players would thus not integrate but perform a two alternative forced choice task. The cutoff also accounts for findings from perceptional cue integration that show a non-linear relationship between cues when cues are too dissimilar [see robust weak fusion (Maloney and Landy, 1989)].

In general, we distinguished two model families; first, models where participants’ decisions were BO or modified BO and, second, models where participants decided using an (unknown) heuristic. We had already tested this set of models in a purely behavioral pilot study with 23 participants and the same experimental setup where 18 out of 23 participants used a (modified) BO strategy. This pilot was then used to derive our hypotheses about the possible model space. An additional behavioral control was conducted to ensure that results were robust to the actual presence/absence of partners with regard to the integration of social and individual information (see ESM).

### BO models

This model family describes a BO strategy derived from work on the integration of information in multi-modal perception tasks (Alais and Burr, 2004). The deviation of final from initial choice is weighted (*ω*) by the accuracy of the available information (individual: acc_I_, social: acc_S_) represented in the variance of the deviation between choice and correct location during phase 1. All models are based on a pure BO model where players correctly estimate the accuracy of the social information and decided in a BO manner (equation 1). Here, more weight is given to individual information when the accuracy of the social players is low and vice versa. For the three integration conditions, *ω* changes based on the two information types involved (equations 2–4)
(1)
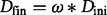

(2)
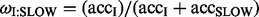

(3)
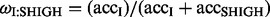

(4)
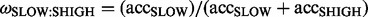



#### BO with individual and/or social error (BO_mod_)

We extended the BO model to allow for the possibility that players estimated their own information and/or one of the social players systematically better or worse than the actual accuracy. This led to five additional models where we allowed for a scaling of the accuracy of one social player or the accuracy of an individual player or both. We added one or two free parameters (*k*_s_, *k*_i_ or both) that scaled the social and individual accuracy (equation 1 with equation 5 through 7 for scaling of individual and low accuracy social player). As it is possible that players misjudged either of the social players, we calculated two models where *k*_s_ either modified the low accuracy social player or the high accuracy player.
(5)
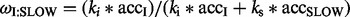

(6)
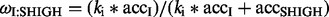

(7)
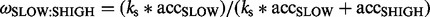



For modeling our fMRI data (discussed later), we chose the *k*_i_ from the BO model with an accuracy adjustment *k*_s_ for the high accuracy players (see ESM). That is, *k*_s_ will enter in equation (6) and not into equation (5).

### Non-BO strategies

#### Accuracy independent deviation

Players engaging in this strategy will take the distance between the two choices into account (*D*_fin_), but not the different accuracies of each player. This leads to higher deviations with higher distance between the two choices independent of the accuracy (equation 8). This model is equivalent to a linear regression modeling one slope for the relationship between *D*_ini_ and *D*_fin_ and omitting the intercept.
(8)
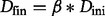



#### Full model with three independent slopes

Some players reacted in all three conditions in a different manner which made it difficult to ascertain which strategy they used. This model captures this possibility and allows for three different slope parameters (*β*) for each condition *i* (equation 9) again omitting an intercept.
(9)
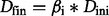



Model selection was based on Bayesian information criterion (see ESM for details). To link our modeling results to our fMRI analysis, we entered the inverse of *k*_i_ as a regressor in our 2nd level fMRI random effects analysis. As we expected larger differences between the purely social information (*S*:*S*) and mixed information type conditions (*I*:*S*) for players neglecting social information, this transformation ensured that high values entered in our random effects analysis were associated with players who underexploited social information. We had no specific *a priori* hypothesis about the effect of *k*_s_ and decided against an exploratory analysis of correlated brain activations with different levels of *k*_s_.

## RESULTS

**Fig. 2:**
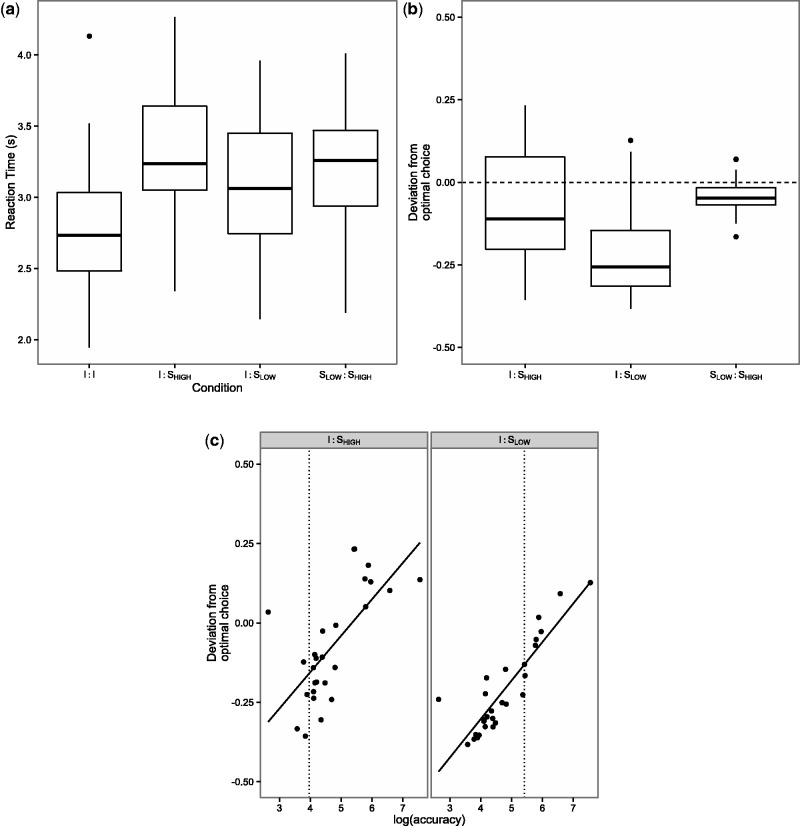
(**a**) Players reacted faster in the *I*:*I* condition. A linear mixed model with error structure based on a gamma distribution with condition and the distance players moved the circle for their second choice as fixed effects and player identity as random effect on the intercept showed a significant difference between the *I*:*I* and other conditions (contrast *I*:*I* < *I*:*S*_Low_: *z* = 14.43; *P* < 0.001; *I*:*I* < *I*:*S*_High_: *z* = 9.87; *P* < 0.001; *I*:*I* < *S*_High_:*S*_Low_: *z* = 10.96; *P* < 0.001; *n* = 29). (**b**) Players deviated from BO choice in the conditions that included individual information. In contrast, they responded near BO in the condition where social information alone was presented. Deviation from BO choice was determined by *D*_BO_ = 0.5 − [(slope_REG_)/(slope_BO_ + slope_REG_)], with slope_REG_ derived from a linear regression for each condition (*β*_i_ from equation 8) and slope_BO_ calculated from the corresponding accuracies (*ω* from equation 1) (*n* = 29). Boxplots in (**a**) and (**b**) show median and box ranges from first to third quartile. Whiskers cover an additional 1.5 inter quartile range each. All data points outside this range (outliers) are represented as black dots. (**c**) Players deviate stronger from BO choice in the *I*:S conditions when they had achieved high accuracy scores in phase 1 (Correlation between deviation from BO choice in phase 2 and accuracy in phase 1 via Kendall Rank correlation: *I*:*S*_HIGH_: *T* = 273, *P* = 0.008, *τ* = 0.34; *I*:*S*_LOW_: *T* = 348, *P* < 0.001, *τ* = 0.71, *n* = 29). Left panel depicts *I*:*S*_HIGH_ condition, right panel *I*:*S*_LOW_ condition. Vertical lines illustrate the accuracy of the two social players.

Players exploited information provided in phase 2 to increase their accuracy (variance of deviation from the correct location) compared with phase 1 [phase 1: median ± median adjusted deviation (MAD)* = *80.56 ± 50.52; phase 2: median ± MAD = 53.81 ± 23.18; Wilcoxon-signed rank test: *V* = 407, *n* = 29, *P* < 0.001]. Reaction times between conditions were significantly lower in the *I*:*I* condition, but only by 0.5 s (Figure 2a). For each individual player, we derived the BO response from their own accuracy during phase 1 and the social players’ accuracy scores (equation 1). We then fitted separate linear regression to each integration conditions (i.e. *I*:*S*_LOW_, *I*:*S*_HIGH_, *S*_LOW_:*S*_HIGH_; equal to equation 9) with the difference between the two stimuli on the circle as independent and the distance between choice and reference stimulus after the decision as dependent variables. When comparing these two measures, we found that players deviated little from BO choice in the *S*_LOW_:*S*_HIGH_ condition, whereas the majority of players underused social information in the *I*:*S* conditions (Figure 2b). The suboptimal use of social information was correlated with player’s own accuracy in phase 1 providing evidence that confidence in individual information was indeed modulated by players’ estimation of their accuracy in phase 1 (Figure 2c).

### Model results

For most players (*n* = 20), one model from the BO model set was supported by the data over and above models based on non-Bayesian strategies (BIC weights: BO models: mean ± s.d. = 0.67 ± 0.4; accuracy independent deviation: 0.12 ± 0.3; full model with three independent slopes: 0.2 ± 0.34). In eight cases, however, BO models were not supported unequivocally, e.g. when players used a heuristic that involved always placing their decisions at the midpoint between the two circles, independent of condition. These players were excluded from the subsequent fMRI analysis because their strategy deviated from that of other players in a manner that precluded their contribution to addressing our main imaging questions. One additional player was excluded because choices in the *I*:*I* condition were not consistently placed in the middle between the two stimuli. For the other players, we derived the estimate how strongly they modified their own accuracy of *k*_i_ from equations (5) and (6). In general, models confirmed that players underused social information when integrating social and individual information with *k*_i_ values significantly below one (Median ± MAD = 0.78 ± 0.56; Wilcoxon signed rank test; *V* = 128, *P* = 0.041, *n* = 29). Here, low *k*_i_ values denoted a bias for individual information. As we were interested in a measurement of how strongly players favored individual information, we used the inverse of *k*_i_ (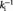) as a covariate in our random effects fMRI model. This led to high values denoting a strong neglect of social information. The appropriateness of our choice of covariate is further supported by the strong correlation between 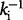 and players’ accuracy scores in phase 1 (Kendall’s Rank correlation: *T* = 282, *P* = 0.002, *τ* = 0.39, *n* = 29). The model estimate for the bias for social information (*k*_s_) was not significantly different from one (median ± MAD = 1.0 ± 0.24; Wilcoxon-signed rank test; *V* = 221, *P* = 0.95, *n* = 29). That is, our model results uphold our observations that players decided near BO in the *S*:*S* condition.

### fMRI results

A comparison of activations associated with exploitation of individual information alone (*I*:*I*) and conditions requiring integration of information (*I*:*S* and *S*:*S*) revealed significant activation in precuneus, right medial parietal lobe, dorsomedial prefrontal cortex, left dorsolateral and bilateral ventrolateral prefrontal (VLPFC) regions (Table 1). There were no significant differences above the threshold criterion in activation between the *I*:*S* condition and the *S*:*S* condition indicating that similar processes were involved in both conditions.

**Table 1:** Conditions involving social information showing greater activation than the individual information alone condition (*P* < 0.001 on voxel level and *P* < 0.05 FWE error corrected on cluster level

Cluster		Peak				Regions	
*P* FWE-corr	Cluster size	*T* statistic	*x*	*y*	*z*	L/R	
<0.0001	3159	6.68	8	−66	38	R	Precuneus
		6.30	2	−58	44	R	Precuneus
		5.73	6	−54	60	R	Precuneus
0.00036	649	6.48	−38	22	22	L	IFG (p. Triangularis)
		5.24	−30	24	4	L	Insular cortex
		5.10	−34	18	8	L	Insular cortex
0.01529	317	6.10	52	−62	14	R	Middle temporal gyrus
0.04154	242	5.71	−46	−70	16	L	Middle occipital gyrus
		5.31	−40	−76	18	L	Middle occipital gyrus
		4.33	−34	−70	22	L	Middle occipital gyrus
<0.0001	1807	5.62	10	10	52	R	SMA
		5.20	36	16	24	R	IFG (p. Triangularis)
		5.17	44	28	26	R	IFG (p. Triangularis)
<0.0001	793	5.38	−32	−2	54	L	Precentral gyrus
		4.86	−22	0	62	L	Superior frontal gyrus
		4.52	−42	0	36	L	Precentral gyrus
0.00064	593	5.22	30	26	2		
		5.10	30	40	12		
		5.09	32	42	20	R	Middle frontal gyrus

Coordinates in MNI space. SMA, supplementary motor area.

**Fig. 3:**
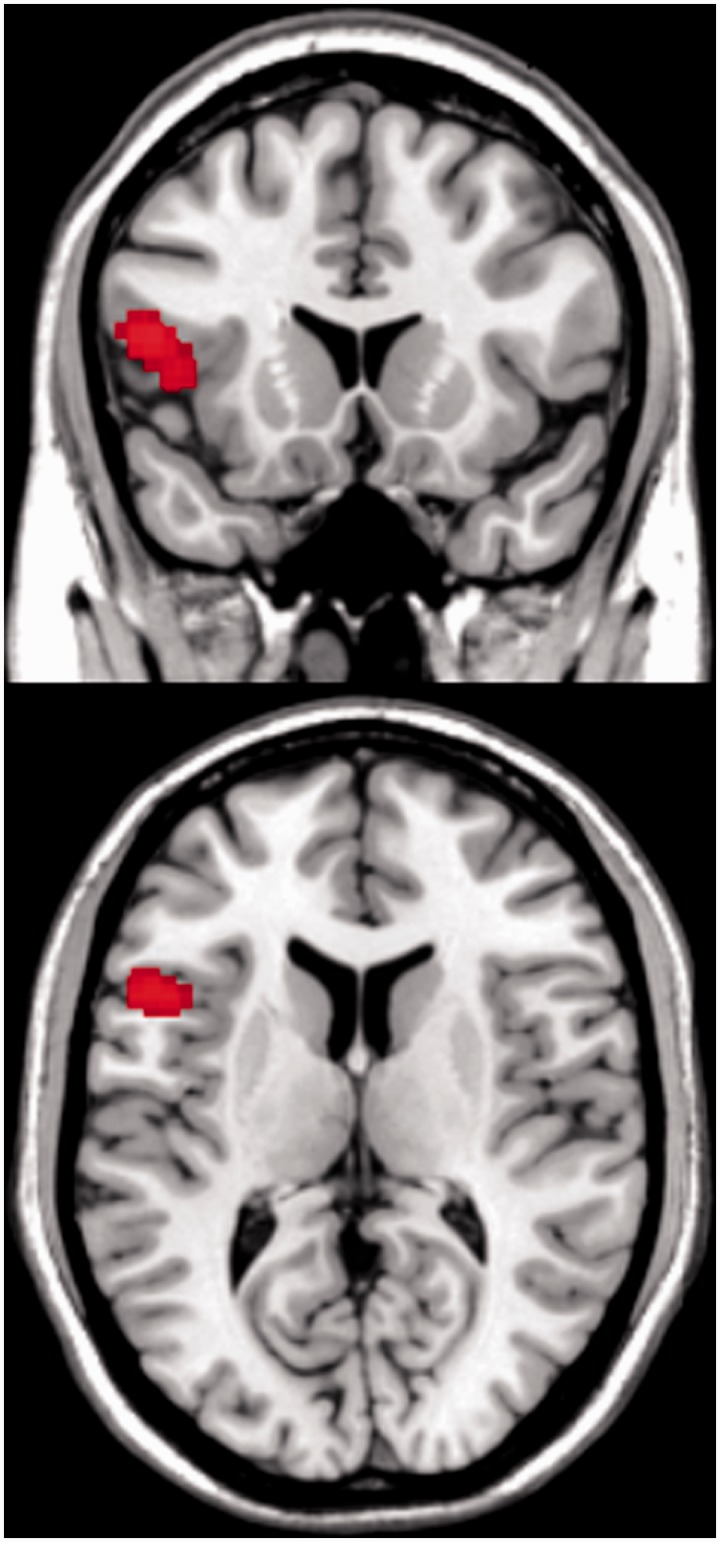
Players who neglected social information (high 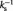) exhibited larger differences in lIFG activation in the *S*:*S* > *I*:*S* contrast (MNI position of depicted slices: *x* = −49, *y* = 17, *z* = 11; scale represents *t*-values).

As we were primarily interested in the neural substrate moderating the integration of individual and social information, we entered our estimate for how strongly individuals relied on individual information (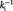) as a covariate into the contrast *S*:*S* > *I*:*S*. Here, we found a significant positive correlation with the level of differential activation in the left inferior frontal gyrus (lIFG). That is, players with a relatively lower activation in the lIFG showed less efficient integration of social and individual information. One local maximum located to the pars triangularis (MNI: −44 20 2; cluster size: 299; *P* = 0.033; FWE corrected) and a second local maximum located to pars opercularis (MNI: −52 14 14) (Figure 3). Note that this entire region showed a partial overlap with the VLPFC, we found for the contrast between *I*:*I* and the integration conditions (*I*:*S* and *S*:*S*).

## DISCUSSION

Our findings highlight that subjects use a *copy-when-uncertain* strategy, a fundamental form of social learning in animals (Kendal *et al*., 2005; Galef *et al.*, 2008) and humans (Weizsäcker, 2010; Morgan *et al.* 2012) alike. Information from social sources alone is integrated in a near BO manner with the weight given to each source scaling with its respective reliability. Crucially, an integration of individual information and social information deviates systematically from Bayes optimality, with individual information consistently weighted more heavily across the *I*:*S* conditions. Crucially, the over-proportional weight given to individual information scaled with the accuracy of individual information.

We deliberately deviated in our experimental design from 2AFC tasks like informational cascades experiments (Bikhchandani *et al*., 1992) where individual skill differences are minimal or not task relevant. This enabled us to model the relationship between uncertainty, variance of player decisions, and social information use to reveal a clear relationship between the two in individual decisions. This emphasis on individual non-joint decision making distinguishes our approach from those that depend on verbally expressed confidence statements that explicitly address metacognitive abilities (Bahrami *et al*., 2010). Importantly, we established that deviation from Bayes optimality is not due to task-specific constraints because players integrated in a near BO manner when presented with information from two social players. Moreover, a control experiment that involved direct interaction of players and actual information from these players showed behavioral results matching the findings from the fMRI experiment (see ESM). That is, subjects in the scanner reacted similarly to situations involving direct social interaction.

At the level of brain function, we identified patterns of activation between a control condition (*I*:*I*) and conditions requiring integration of two information sources that are engaged in social decision-making tasks and in the exertion of cognitive control. For example, the precuneus is implicated in self-referential processing when comparing oneself with others (Cavanna and Trimble, 2006) or when taking a third person perspective (Vogeley *et al*., 2004). The dorsomedial prefrontal cortex is involved in social cognition across various tasks (Amodio and Frith, 2006) while VLPFC and DLPFC are linked to executive control of behavior (Tanji and Hoshi, 2008). At the same time, there is no above threshold activation in areas typically associated with mentalizing about others, for example the right temporoparietal junction (Van Overwalle and Baetens, 2009). This raises the question as to whether participants would have reacted differently when confronted with computer-generated information instead of real participants’ decisions. Differences in behavior and neuronal activation between conditions with human or computer agents arise when participants make inferences about the goals of their partner (Rilling *et al*., 2004). In our experiment, participants process observational information that was obtained inadvertently without a signaling intention of their partner or need to infer such an intention. In such cases, there is no activation difference between conditions with a computer or human actor with regard to the brain regions identified in our basic contrast (Chaminade *et al*., 2012). Moreover, individuals often use strategies toward a computer controlled agent that are similar to those in real social contexts (Nass and Moon, 2000). It is thus likely that a computer control would not yield qualitatively different results.

To ascribe functional specificity to the observed brain activation patterns, we adopted the strategy of exploiting individual differences in player accuracy that arose out of varying systematic uncertainty estimates between individuals. As all players integrated two social information sources in an almost BO fashion, we expected less engagement of regions responsible for the integration process in the *I*:*S* relative to the *S*:*S* condition for players who neglected social information (high individual accuracy). This allowed us to identify lIFG as the sole region modulated by the degree that players overvalued their own information relative to the social information. Similar levels of activity were seen in this region when players integrated individual and social information in an optimal manner while for players who neglected social information there was less engagement of this region. This suggests that this region exerts a regulatory role wherein greater engagement is associated with increasingly efficient integration of social and individual information. This fits with its known involvement in processes such as control of memory retrieval or cue conflict in working memory (Badre and Wagner, 2007) and specifically for representing the ambiguity of a cue (Bach *et al*., 2009). This region is also implicated in response inhibition, evident for example in patients with lesions to this regions who manifest impaired performance in go/no go tasks (Swick *et al*., 2008). Moreover, the IFG in conjunction with the subthalamic nucleus is critical for instrumental control to overrule Pavlovian influences (Guitart-Masip *et al*., 2012).

In our study, the IFG is engaged if individual information is not overvalued relative to social information where it may exert an inhibitory influence on responses based on individual information alone. Thus, engagement of this region may allow participants to appropriately exploit information from other sources so as to make an improved, near optimal choice. This interpretation is consistent with findings for a mediating role of the lIFG in control processes when subjects need to decide between healthy and unhealthy food options (Hare *et al*., 2009). One upshot is that the brain areas we highlight as being recruited for integration of social information are in fact components of a network that is fundamental to decision-making under uncertainty (Platt and Huettel, 2008; Bach and Dolan, 2012).

In conclusion, we show that individuals combine distinct sources of social information in a Bayes optimally manner, but when required to integrate individual and social information they are biased toward a reliance on individual information when the latter is highly accurate. In other words in the latter context, there is a strong prior on individual information. Theoretical work on decision-making under uncertainty shows strong priors are to be expected when the cost for making a mistake outweighs the benefit of improving the decision (Trimmer *et al*., 2011). In our case, the benefits in refining a decision when an individual decision is already accurate are comparably low, such that taking social information into account carries unnecessary risks. This accords with theoretical accounts on the evolution of social learning where social information, even though available at no cost, embodies a degree of hazard such as the risk of being outdated in variable environments (Kameda and Nakanishi, 2003; Rieucau and Giraldeau, 2011). The intriguing possibility is that humans have evolved stronger priors for individual than social information because a slight overestimation of individual skill and prowess is highly adaptive across a wide range of conditions (Johnson and Fowler, 2011).

## Supplementary Material

Supplementary Data
