# Supplementary material for: The neural underpinnings of an optimal exploitation of social information under uncertainty
Source: Soc Cogn Affect Neurosci. 2013 Dec 2;9(11):1746–53. doi: 10.1093/scan/nst173 (PMC4221218; doi:10.1093/scan/nst173)
Supplement: Supplementary Data [file supp_9_11_1746__index.html]

The neural underpinnings of an optimal exploitation of social information under uncertainty — The neural underpinnings of an optimal exploitation of social information under uncertainty — Supplementary Data 

# The neural underpinnings of an optimal exploitation of social information under uncertainty

## Supplementary Data

files

**Files in this Data Supplement:**

- Supplementary Data - pdf file
