# Supplementary material for: The neural underpinnings of an optimal exploitation of social information under uncertainty
Source: Soc Cogn Affect Neurosci. 2013 Dec 2;9(11):1746–53. doi: 10.1093/scan/nst173 (PMC4221218; doi:10.1093/scan/nst173)
Supplement: Supplementary Data [file supp_nst173_scan-13-265-File006.pdf]

Electronic Supplementary Material accompanying *The neural underpinnings of an optimal exploitation of social information under uncertainty.*

Toelch, Bach & Dolan

Model selection

We fitted all models to individual data using the port algorithm of the optimize function in R 2.15.2 with starting values for both k parameters of 0.5, restricting k to positive values. For the AID and FM model we used the lm function in R for parameter estimation. From the maximum log likelihood (ℓ) of each model we calculated the Bayesian Information Criterion (BIC) (Burnham & Anderson, 2002) (Equation S1 with m giving the number of free parameters and n the number of data points). We selected the model with the lowest BIC from each family. If the lowest BIC of the non Bayesian model family was at least 2 points lower than the lowest BIC of the Bayes Optimal model family we excluded this player from the fMRI analysis. This conservative criterion ensured that we did not include payers whose brain activation patterns stemmed from implementation of a radically different cognitive process. For the rest of the players we calculated a model with both k_i and k_s, choosing the k_s for the social player from the model with the lower BIC. For the fMRI analysis this was the BO model that assumed that players modified the acc_{Shigh}. This model had the lowest average BIC weight and there was no model with k_s influencing acc_{Slow} that had a lower BIC than the model of choice.

$$BIC = 2 * \ell + m * ln(n) \tag{S1}$$

Direct social interaction control

In the fMRI experiment participants did not directly meet the other players before the experiment and thus might have reacted differently than if the provided information stemmed from an actually present player. To rule out this possibility we tested 9 additional subjects in a modified, two phase version of the above design. Here, subjects played with present and visible players that were introduced to each other at the beginning of the session. Subjects saw the actual choices of one of the other players as information during the game. In the first phase (75 trials) players saw, after making a guess, their own choices, the choices of one and always the same person in the room, and the correct location of the stimulus. In the second phase (75 trials), players saw their own choices from the first phase paired with the choice of the other player.

We found the same effect that players underused social information in the control experiment where subjects directly interacted with other players. Players decisions deviated from Bayes optimal choice (Deviation calculated as in Figure 2c: $Mean \pm SEM = -0.17 \pm 0.05$). Here, as in the main experiment, the degree to which players neglected social information was also positively correlated with players own accuracy (Kendall Rank correlation: $T = 34, p < 0.001, \tau = 0.88; n = 9$).

Table S1: Parameter estimates of the BO model with k_s influencing acc_{Shigh}. Raw BIC values are displayed for the three model classes in the last three columns. Asterisked players were excluded from fMRI analysis. Players with one asterisk were excluded due to a non BO model having the lowest BIC. The player with two asterisks was excluded due to his decisions in the $I : I$ condition shedding serious doubts whether he understood the game correctly.

id	k_i	k_s	BO	AID	FM
1	1.31	0.68	708.96	1007.30	708.85
2	0.15	1.01	632.04	867.67	637.08
3	0.11	1.00	357.75	968.54	362.06
4	0.87	1.03	624.51	843.28	628.96
5	0.79	0.81	992.90	1072.24	1006.44
6	0.36	1.10	605.59	891.69	596.88*
7	0.32	0.14	1129.79	1278.98	1120.12*
8	1.02	0.92	690.94	928.27	702.54
9	1.10	1.00	672.01	1002.72	681.03
10	2.01	0.99	712.72	869.62	718.44
11	0.36	1.15	1112.70	1120.85	1121.38**
12	0.42	0.48	863.11	1012.93	869.12
13	5.36	2.21	862.58	923.48	800.18*
14	0.41	0.81	1002.73	1109.77	1007.69
15	0.22	0.82	815.11	949.47	817.60
16	1.09	1.17	838.54	970.54	848.02
17	1.48	1.10	849.05	995.03	859.35
18	0.67	0.60	1087.94	1194.72	1094.87
19	1.10	1.02	508.56	807.96	515.38
20	0.44	1.69	831.58	925.02	833.23
21	0.87	1.05	695.41	971.72	704.25
22	1.44	1.29	1218.31	1216.91	1226.49*
23	0.03	0.97	566.52	561.83	570.74*
24	0.40	1.07	952.34	954.62	958.51
25	1.60	1.19	980.31	975.47	984.37*
26	0.78	0.65	865.68	1002.38	868.12
27	0.78	1.00	614.32	782.10	619.44
28	0.07	0.98	671.21	667.10	675.91*
29	0.69	1.35	739.26	936.36	707.46*

Instructions for participants

Localise it! Part 4

Instructions

In this experiment you will make decisions. The experiment is divided into three phases and each phase consists of several rounds. Each round your aim is to relocate the position of a white disc that had briefly appeared on your computer screen as accurately as possible.

Phase 1 (outside MRT): When you start the game you will be prompted to start the round by pressing a key on your keyboard (please avoid pressing special keys like ESC, F1 through F12). After this you will see a small circle in the middle of the screen. The mouse pointer will be moved from the center by the computer and your task is to keep the mouse pointer inside the small circle. After a couple of seconds a white disc will be briefly flashed along the circuit of a larger circle. Next a short time interval follows where you can see several non-relevant white discs randomly appearing. Keep on holding the mouse pointer in the inner small circle.

Next you will be able to see the outer circle and your task is to move your mouse pointer as close as possible to the location, where you think the white disc appeared (the center of the disc is the criterion). A small red disc marks your choice and will become visible when your mouse pointer approaches the outer circle. If you are satisfied with your choice, confirm by pressing the left mouse button. In the first phase (40 rounds) you will have to guess the position of the white disc as exactly as possible (similar

to the second phase). You will then receive feedback (a small white disc), showing where the white disc really was. With an additional mouse click you will end the round. This phase is intended for you to familiarize with the controls and practice your accuracy.
Phase 2 (outside MRT): The second phase (120 rounds) is similar to the first phase. Again you will have to localize the position of the disc. In addition to your own guess, you will see the guesses of two other players (displayed as green and yellow disc). These players have previously played the same game in a pilot study and have the same amount of experience as you (played 40 rounds in the first phase). The colours are dedicated to one particular player and do not change during the game. Additionally, you will receive feedback on the real location of the white disc (displayed as small white disc) to estimate your own and the other players accuracy. Sometimes the guesses can overlap when you and another player have guessed similar locations. In this case the guesses will be displayed slightly shifted. We recommend already thinking of a strategy for phase 3 in this phase.
Phase 3 (inside MRT): In the third phase (2 times 120 rounds) you can no longer see the flashing white disc at the start of each round. Instead you will see two guesses from the second phase after 2 seconds and have to localize the white disc. That is, you will see guesses from the second phase (your own and/or other players guesses) and based on these guesses have to make a further guess on where the disc appeared without actually seeing the white disc again. The position of the disc is of course the same as in the second phase. The colours of the guesses are also the same as in Phase 2, i.e. red for your own guesses and green and yellow for the other players guesses. You will receive only two of these guesses, for example your own guess (red) and the guess of the green player or in a different round the guess of the green player and the yellow player. You have to pay attention at the beginning of each round which guesses are available.
In 60 rounds in the third phase both guesses will be displayed in red. This means that both guesses were created by yourself. This is possible since in the second Phase two rounds (randomly distributed) were paired in which the white disc appeared in the same location. The two red guesses come thus from two different rounds with the white disc in the same location. The order in the third phase was randomized and is not the same as in the second phase. Some rounds from phase 2 are selected several times but with different combinations of guesses.
After the experiment, you will receive information about your total points. The more accurate your guesses were for each guess, the more points you will receive. You can collect most points in the third phase, so decide with care. Should you not keep the mouse pointer at the beginning of each round in the small circle (first and second Phase), the circle will turn red and you will lose points. This loss is significant if the mouse pointer is constantly outside the circle. The more points you have gathered the more money you will receive at the end of the experiment. You will receive a basic remuneration of 8 Euro per hour and a variable bonus of up to 9 Euro.
After the experiment we would like you to fill in an additional questionnaire. We expect the experiment to take slightly more than two hours. You may ask questions before the experiment and while outside the scanner. Within the MRT we kindly ask you to avoid all unnecessary head movement since this will lead to noisy signals that could possibly render the collected data useless.

Thank you for participating,
The experimenters

References

Burnham, K. P., & Anderson, D. (2002). Model Selection and Multi-Model Inference (2nd ed.). Springer.
